# 30-Day Complications After Day-Case Versus Inpatient Ureterorenoscopy: A Retrospective Cohort Study

**DOI:** 10.3390/jcm15093391

**Published:** 2026-04-29

**Authors:** Roos Van Slagmaat, Esther Pita Marieke Duindam, Jessica Steenbruggen, Diederick Duijvesz

**Affiliations:** Department of Urology, Canisius Wilhelmina Hospital Nijmegen, 6532 SZ Nijmegen, The Netherlands; roosvanslagmaat99@gmail.com (R.V.S.); je.steenbruggen@cwz.nl (J.S.)

**Keywords:** ureterorenoscopy, urolithiasis, day-care, complications

## Abstract

**Background**: Rising healthcare demands and the impact of the COVID-19 pandemic have prompted a transition from inpatient to day-case ureterorenoscopy (URS) to reduce costs and optimise bed capacity. Although international studies support the safety of day-case URS, evidence from The Netherlands is limited. Furthermore, Dutch and European urological guidelines do not provide explicit recommendations for standardising URS as a day-case procedure. The present study compares 30-day complication rates of URS between the pre-COVID-19 era (2019) and current practice (2023). **Methods**: A retrospective cohort study was conducted, including all patients who underwent elective URS at Canisius Wilhelmina Hospital, the Netherlands, in 2019 and 2023. Patients under 18 years of age, as well as those undergoing emergency procedures and combined procedures, were excluded. The primary outcome was the occurrence of complications within 30 days, classified according to the Clavien–Dindo system. Secondary outcomes included identification of clinical and procedural predictors of complications. Statistical analysis was performed using SPSS and included Chi-square tests, *t*-tests, Mann–Whitney *U* tests, and multivariable logistic regression. **Results**: Of 619 screened patients, 495 were included: 230 patients in 2019, 265 patients in 2023. Baseline characteristics were comparable between groups. The overall complication rate was low (15.7% in 2019, 20.0% in 2023) and did not differ significantly between day-case and inpatient URS (*p* = 0.209). In multivariable logistic regression, day-case URS was not associated with an increased risk of complications (OR = 0.91, 95% CI 0.46–1.81, *p* = 0.795). There were no other significant predictors of complications. **Conclusions**: The findings suggest that day-case ureterorenoscopy may be a safe and feasible approach and may offer opportunities for cost savings, as no increase in postoperative complications was observed compared with inpatient procedures. However, these results should be interpreted with caution given the observational design.

## 1. Introduction

Nephrolithiasis and urolithiasis are among the most common urological disorders in Europe [1]. When intervention is necessary, ureterorenoscopy (URS), extracorporeal shockwave lithotripsy (ESWL), and percutaneous nephrolithotomy (PNL) are used, depending on the case [2]. At Canisius Wilhelmina Hospital (CWZ), URS is performed most frequently, with around 300–350 cases annually [3]. Traditionally, URS patients were admitted for at least one postoperative night to monitor for potential complications such as pain, haematuria, or fever. However, increasing healthcare costs and hospital bed pressures have prompted reconsideration of the need for routine postoperative admission following uncomplicated URS. The average cost of an additional inpatient day is approximately €514, representing an estimated 20% increase in total patient-related costs [4]. Notably, neither the Dutch Association of Urology [5] nor the European Association of Urology [6] currently provides explicit recommendations on standardising URS as a day-case procedure. Several international studies have demonstrated that day-case URS is both safe and feasible. A prospective cohort study including 544 patients found that 78% were discharged on the day of surgery [7]. Other studies reported complication rates between 3% and 6% following day-case URS [8,9]. In line with this evidence, CWZ implemented same-day URS as the standard of care in 2023. Despite these encouraging results, national data on the safety and feasibility of day-case URS in the Netherlands remain scarce. Moreover, the influence of patient-, stone-, and procedure-related factors on postoperative outcomes has not been comprehensively evaluated. A single-centre comparison of inpatient versus day-case URS may therefore provide valuable insights into optimal patient selection and perioperative management. The present study aimed to evaluate the safety and feasibility of day-case ureterorenoscopy (URS) at CWZ by comparing outcomes between 2019 (pre-COVID-19) and 2023 (current practice). To capture the shift in clinical practice, these two years were deliberately selected: in 2019, prior to the COVID-19 pandemic, most URS patients were routinely admitted for overnight observation, whereas by 2023, in response to increasing healthcare demands and the need to optimise hospital bed capacity, day-case URS had become the standard of care. The primary endpoint was the incidence of postoperative complications within 30 days, classified according to the Clavien–Dindo system. Secondary analyses assessed patient-, stone-, and procedure-related predictors of postoperative complications.

## 2. Methods

### 2.1. Study Design and Setting

This retrospective observational cohort study was conducted at the Canisius Wilhelmina Hospital (CWZ), a single-centre teaching hospital in Nijmegen, The Netherlands. All patients (≥18 years) who underwent elective URS during two predefined study periods, 1 January to 31 December 2019 and 1 January to 31 December 2023, were included.

All URS were performed by experienced urologists or by residents in urology under close supervision. Depending on stone characteristics and anatomical considerations, either semirigid or flexible URS, or a combination of both, was used. Choice of instrument type followed standard institutional protocols. An Olympus (URF-V3 or OES ELITE) or PUSEN URS was routinely used for procedures. A double-J ureteral stent was placed at the discretion of the surgeon.

Complications were classified according to the Clavien–Dindo grading system. This system grades postoperative events from grade I (minor deviation without intervention) to grade V (death) based on the level of therapeutic intervention required. All postoperative complications occurring within 30 days were systematically recorded, including minor events classified as Clavien–Dindo grade I, to ensure comprehensive outcome assessment. Due to the retrospective design, detailed categorisation of specific complication types was not consistently available.

The study protocol was approved by the institutional review board of Canisius Wilhemina Hospital Nijmegen (protocol code 2024-0018, 30 April 2024), and the requirement for informed consent was waived owing to the study’s retrospective design.

### 2.2. Study Population

Eligible patients were those who underwent elective URS for urolithiasis (renal or ureteral stones) or suspected urothelial carcinoma. Exclusion criteria were: age < 18 years, URS performed in an emergency setting, and URS combined with another surgical procedure (e.g., percutaneous nephrolithotomy).

### 2.3. Data Collection

Data were obtained from the hospital’s electronic medical record system using diagnosis–treatment combination (DBC) registration codes. This automated extraction process minimised selection bias by avoiding manual screening. All eligible cases within the defined periods (2019 and 2023) were consecutively included, representing a full-population analysis.

Complications and relevant clinical variables were identified from electronic records and operative reports. Stone characteristics, including size, location, and density, were determined preoperatively based on computed tomography (CT) scans. Stone size was defined as the largest diameter of the largest stone, and density was expressed in Hounsfield units (HU) measured on CT. Stone location was classified as renal, proximal ureteral, or distal ureteral.

Anatomical anomalies were defined as any congenital or acquired variation that could potentially affect ureteroscopic access, including a narrow ureteral lumen, bifid collecting system, solitary kidney, or other structural abnormalities documented by imaging or operative findings.

Stone-free status was assessed intraoperatively by direct visual inspection and defined as complete absence of calculi or only passable residual fragments.

### 2.4. Statistical Analysis

All data were pseudonymised and entered into a secure Castor EDC database accessible only to the investigator and principal investigator. After verification, data were exported to SPSS (version 29.0.2.0, IBM Corp., Armonk, NY, USA) for analysis.

Descriptive statistics were used to summarise baseline characteristics. Continuous variables were expressed as mean ± standard deviation (SD), and categorical variables as counts and percentages. Between-group comparisons were performed using the Chi-square test for categorical variables, the independent-samples *t*-test for normally distributed continuous variables, and the Mann–Whitney *U* test for non-normally distributed data. Results were reported as odds ratios (ORs) with 95% confidence intervals (CIs). Variables were selected for inclusion in the multivariable logistic regression model based on clinical relevance and prior literature. A two-sided *p*-value < 0.05 was considered statistically significant. *p*-values were reported for statistically significant results, while non-significant findings were indicated as ‘NS’ (*p* ≥ 0.05).

## 3. Results

### 3.1. Study Population

**Figure 1:**
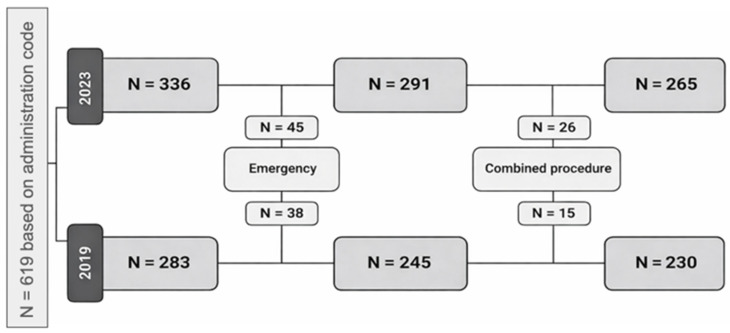
Flowchart of inclusions and exclusions stratified by year.

A total of 619 patients were included based on the administration code. After applying the exclusion criteria, 495 patients remained eligible for analysis, including 230 procedures performed in 2019 and 265 in 2023 (Figure 1).

**Figure 2:**
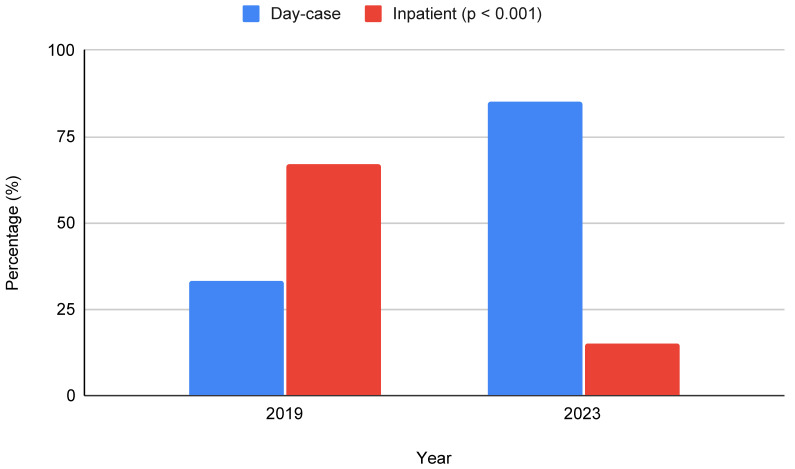
Distribution of admission type stratified by year. A significant increase in day-case URS was observed in 2023 compared to 2019 (Chi-square test, *p* < 0.001).

The proportion of day-case URS increased significantly over time, from 33% (75 patients) in 2019 to 85% (225 patients) in 2023 (*p* < 0.001), reflecting the implementation of same-day URS as standard practice at the institution (Figure 2). In 2023, a total of 40 patients (15%) were still admitted overnight. Reasons for admission were mainly logistical or patient-related rather than clinical, including late operating times, insufficient postoperative pain control at the day-care unit, or pre-arranged admission based on patient preference or prior positive experience with inpatient URS.

### 3.2. Baseline Characteristics

Baseline patient characteristics are presented in Table 1. The mean patient age was comparable between the two years (59 ± 14 vs. 59 ± 15 years). However, the proportion of female patients increased significantly in 2023 compared with 2019 (42% vs. 32%, *p* = 0.016). Body mass index (BMI) and renal function (eGFR) were similar between the cohorts. The prevalence of patients with ASA ≥ 3 did not differ significantly (20% in 2019 vs. 24% in 2023). Likewise, smoking behaviour, anticoagulant use, and α-blocker use showed no significant variation between years.

### 3.3. Procedural Characteristics

The distribution of therapeutic versus diagnostic URS procedures was identical in both cohorts (86% vs. 14%), and laterality (left, right, or bilateral) showed no significant differences (Table 2). Preoperative JJ-stent placement tended to be less frequent in 2023 (33% vs. 40%), while postoperative catheterisation rates remained consistently high (approximately 90%). Regarding stone characteristics, mean stone density was higher in 2023 than in 2019 (1169 ± 372 vs. 1094 ± 375 HU), and mean stone size was comparable between years (8.3 vs. 8.2 mm). A significantly greater proportion of patients presented with anatomical anomalies in 2023 (23% vs. 14%, *p* = 0.013). The stone-free rate was similar across cohorts (79% in 2019 vs. 80% in 2023). Mean operative time showed a nonsignificant trend towards shorter procedures in 2023 (42 ± 24 min) compared with 46 ± 25 min in 2019. Finally, the distribution of operator level differed markedly between cohorts, with residents performing a larger share of procedures under supervision in 2023 (*p* < 0.001).

### 3.4. Postoperative Outcomes

**Figure 3:**
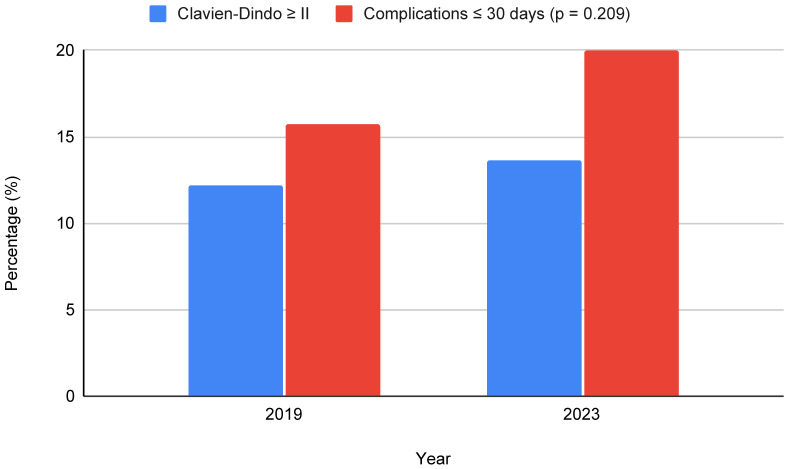
Postoperative outcomes stratified by year (2019 vs. 2023). No statistically significant difference was observed between groups (Chi-square test, *p* = 0.209).

Postoperative complications within 30 days are presented in Figure 3. The overall complication rate was 15.7% (36 complications) in 2019 and 20.0% (53 complications) in 2023, showing no statistically significant difference between years (*p* = 0.209). In 2019, postoperative complications were classified as Clavien–Dindo grade I in 8 patients (22.2%), grade II in 26 patients (72.2%), and grade III in 2 patients (5.6%). In 2023, complications were graded as Clavien–Dindo grade I in 17 patients (32.1%), grade II in 33 patients (62.3%), grade III in 2 patients (3.8%), and grade IV in 1 patient (1.9%). The majority of complications were minor (Clavien–Dindo grade I–II), whereas clinically relevant complications (grade ≥ III) were rare. When considering the overall cohort, complications of Clavien-Dindo grade ≥ II occurred in 12.2% of patients in 2019 and 13.6% in 2023, further illustrating the comparable distribution of more than minor complications between both groups.

### 3.5. Multivariable Analysis

Multivariable logistic regression results are shown in Table 3. No significant association was found between admission type and postoperative complications (OR = 1.09, 95% CI 0.55–2.16, *p* = 0.8). Demographic and clinical parameters, including age, sex, BMI, ASA ≥ 3, anticoagulant use, and smoking status, were not independently associated with complication risk. Higher renal function was weakly associated with increased odds of complications (OR 1.024, 95% CI 1.00–1.05, *p* = 0.046), whereas greater stone density was marginally protective (OR 0.999, 95% CI 0.998–1.00, *p* = 0.033). No other operative variables, including URS type, laterality, operative time, or operator level, were significantly related to postoperative outcomes.

### 3.6. Subgroup Analysis

A subgroup analysis was performed to assess whether complication risk differed between day-case and inpatient URS within each study year. In 2019, inpatients had 1.85 times higher odds of postoperative complications compared with day-case patients, though this difference was not statistically significant (OR = 1.845, 95% CI 0.797–4.274, *p* = 0.152). Similarly, in 2023, inpatients also demonstrated a nonsignificant trend toward higher complication rates (OR = 1.653, 95% CI 0.765–3.571, *p* = 0.201).

## 4. Discussion

This study evaluated postoperative outcomes after ureteroscopy by comparing two institutional cohorts from 2019 and 2023, reflecting a transition from routine overnight admission to same-day discharge as standard practice. Several changes in patient characteristics, operative practice, and perioperative logistics occurred over time and provide essential context for the interpretation of the findings.

Key strengths include the use of two large, real-world cohorts with near-complete follow-up and consistent surgical teams and protocols, enhancing internal validity and allowing meaningful assessment of temporal trends. However, the retrospective design limits causal inference. Stone-free status was assessed intraoperatively rather than using standardised postoperative imaging, which may have led to an overestimation of true clearance rates and limit comparability with studies using imaging-based endpoints. In addition, complications managed outside our institution may not have been fully captured, and generalisability may be limited given our centre’s high procedural volume and established outpatient pathways. Furthermore, complications were recorded according to the Clavien–Dindo classification [6], and detailed categorisation of specific complication types was not consistently available, limiting direct comparison with studies reporting granular complication subtypes. Comparison of two distinct time periods may introduce temporal bias, as changes in surgical practice and perioperative management over time may have influenced outcomes independently of admission strategy. Moreover, procedures were performed by multiple surgeons, including both experienced urologists and supervised residents, which may introduce variability in surgical technique and intraoperative decision-making. Additionally, the inclusion of both stone-related and diagnostic URS procedures introduces a degree of heterogeneity, although the proportion of diagnostic cases was relatively small and unlikely to have materially influenced overall outcomes.

The overall complication rate observed in this study was higher than the 4–8% typically reported in outpatient ureteroscopy series [7,8]. This difference is likely explained by methodological variation rather than inferior outcomes. Prior studies often focused on complications requiring intervention or readmission and may have underreported minor events due to narrower definitions or incomplete follow-up. In contrast, our study applied broad and granular criteria, systematically capturing even mild Clavien–Dindo grade I events, supported by near-complete follow-up. Importantly, the distribution and clinical relevance of complications were comparable to those reported in the literature, with most events being minor and manageable in the outpatient setting.

Importantly, complication profiles did not differ between patients undergoing planned overnight admission and those discharged on the same day. This finding aligns with existing evidence suggesting that routine postoperative observation does not confer additional protection following uncomplicated ureteroscopy. Despite evolving patient mix, imaging practices, and trainee involvement, overall complication rates remained stable across years. Although renal function and stone density were statistically associated with complications, effect sizes were small and unlikely to be clinically meaningful. Given the number of events relative to the included covariates, the multivariable model may be subject to overfitting, and these findings should therefore be interpreted with caution. The observed differences in complication rates may partly reflect variability in surgeon-related factors, including experience and operative technique, rather than the effect of admission strategy alone. In addition, as admission decisions were not randomised, patients selected for overnight admission may represent a higher-risk subgroup, reflecting clinical or procedural complexity. This introduces potential confounding by indication, which should be considered when interpreting comparisons between admission strategies.

Appropriate patient selection remains a key factor in the safe implementation of day-case URS. Previous studies have highlighted the importance of clinical, anatomical, and procedural factors in predicting postoperative outcomes and the likelihood of successful same-day discharge [9,10]. In this context, recent work has explored predictive models and scoring systems incorporating variables such as comorbidity burden, stone characteristics, and intraoperative findings to guide perioperative decision-making [11]. These approaches support a more individualised strategy rather than a uniform admission policy. Our findings are in line with this evolving perspective, suggesting that routine admission may be unnecessary for the majority of patients when appropriate selection criteria are applied.

Several temporal trends merit comment. The higher proportion of women in the 2023 cohort likely reflects natural referral variation, as adjusted analyses showed no clinically relevant sex-related effects. Increased documentation of anatomical anomalies probably reflects improvements in imaging quality and reporting rather than a true increase in prevalence. Shorter operative times in 2023 may relate to technological advances, including newer laser platforms, as well as workflow optimisation. Notably, increased resident involvement did not adversely affect efficiency or outcomes, underscoring the robustness of supervision and operative pathways. The higher prevalence of anatomical anomalies in the 2023 cohort may represent a potential confounder, although it was not independently associated with postoperative complications in multivariable analysis.

The broader implications of adopting routine same-day ureteroscopy extend beyond clinical safety. While reimbursement in our healthcare system is identical for inpatient and outpatient management, this is not universal. In some systems, financial incentives favour overnight admission, potentially limiting adoption of outpatient pathways despite evidence supporting safety and cost savings. These considerations may influence institutional decision-making and warrant attention at a policy level.

Although this study includes a substantial sample, larger prospective multicentre studies with standardised outcome definitions and uniform discharge criteria are needed to better identify whether specific patient subgroups may benefit from planned postoperative observation.

## 5. Conclusions

Day-case URS was implemented as standard practice at our institution without an observed increase in postoperative complications, in this cohort, although these findings should be interpreted with caution given the observational design and potential for residual confounding. Routine overnight admission was not associated with reduced postoperative risk and may be unnecessary in selected patients following uncomplicated URS. Broader adoption of same-day URS could yield substantial cost savings, although alignment of national reimbursement models will likely be required to support widespread implementation. Future multicentre and prospective research should further refine patient selection and optimise protocols for day-case URS.

## Figures and Tables

**Table 1:** Baseline patient characteristics stratified by year.

	2019 *n* = 230	2023 *n* = 265	Total *n* = 495	*p*-Value *
Age (in years)	58.9 ± 13.5	59.0 ± 14.8	59.0 ± 14.2	NS
Sex: male or female, (*n*)	157 (68.3%) 73 (31.7%)	153 (57.7%) 112 (42.3%)	310 (62.6%) 185 (37.4%)	0.016
BMI in kg/m^2^	27.9 ± 4.7	28.4 ± 6.4	28.2 ± 5.6	NS
ASA ≤ 2, (*n*) ASA ≥ 3, (*n*)	184 (80.4%) 45 (19.6%)	201 (76.1%) 63 (23.9%)	385 (78.1%) 108 (21.9%)	NS NS
eGFR in mL/min/1.73 m^2^	72.3 ± 17.4	73.3 ± 17.4	72.8 ± 17.4	NS
Smoking, (*n*)	62 (27.2%)	67 (29.5%)	129 (28.4%)	NS
Anticoagulantia, (*n*)	52 (22.6%)	75 (28.3%)	127 (25.7%)	NS
Alpha blocker preop, (*n*)	21 (9.1%)	21 (7.9%)	42 (8.5%)	NS

* *p* < 0.05, NS = not statistically significant (*p* ≥ 0.05). Abbreviations: BMI, body mass index; ASA, American Society of Anesthesiologists; eGFR, estimated glomerular filtration rate. Statistical tests: Chi-square test for categorical variables; independent-sample *t*-test or Mann–Whitney *U* test for continuous variables.

**Table 2:** Procedural characteristics.

	2019 *n* = 230	2023 *n* = 265	Total *n* = 495	*p*-Value *
Therapeutic or diagnostic, (*n*)	197 (85.7%) 33 (14.3%)	227 (85.7%) 38 (14.3%)	424 (85.7%) 71 (14.3%)	0.998
Side: left, right, or combined, (*n*)	116 (50.4%) 111 (48.3%) 3 (1.3%)	144 (54.3%) 115 (43.4%) 6 (2.3%)	260 (52.5%) 226 (45.7%) 9 (1.8%)	0.386 0.278 0.001
Preop JJ-cath, (*n*)	93 (40.4%)	88 (33.2%)	181 (36.6%)	0.096
Postoperative catheter, (*n*)	210 (91.3%)	233 (87.9%)	443 (89.5%)	0.221
Stone density in HU	1093.7 ± 374.8	1169.0 ± 371.7	1137.2 ± 374.4	0.066
Anatomical anomaly, (*n*)	32 (13.9%)	60 (22.6%)	92 (18.6%)	0.013
Stone-free, (*n*)	151 (78.6%)	179 (80.3%)	330 (79.5%)	0.430
Stone size in mm	8.3 ± 3.6	8.2 ± 3.4	8.3 ± 3.5	0.781
Stone in kidney, Proximal or distal, (*n*)	91 (47.4%) 66 (34.3%) 35 (18.2%)	103 (46.4%) 78 (35.2%) 41 (18.5%)	194 (46.9%) 144 (34.8%) 76 (18.4%)	0.874 0.857 0.938
Operative time in minutes	46.3 ± 24.5	42.2 ± 23.6	44.1 ± 24.1	0.061
Operator: UIT, urologist, or combined	8 (3.5%) 166 (72.2%) 56 (24.3%)	38 (14.3%) 178 (67.2%) 49 (18.5%)	46 (9.3%) 344 (69.5%) 105 (21.2%)	<0.001 0.228 0.112

* *p* < 0.05, NS = not statistically significant (*p* ≥ 0.05). Abbreviations: preop JJ-cath, preoperative use of double J-catheter; HU, Hounsfield units; UIT, urologist in training. Statistical tests: Chi-square test for categorical variables; independent-sample *t*-test or Mann–Whitney *U* test for continuous variables.

**Table 3:** Logistic regression: predictors of complications.

	OR	95% CI	*p*-Value *
Day-case Inpatient	0.914 Reference	0.463–1.810 –	0.795 –
Age	1.009	0.980–1.039	0.541
Sex	0.966	0.478–1.952	0.923
BMI	1.038	0.983–1.097	0.180
ASA ≥ 3	0.755	0.290–1.965	0.565
eGFR	1.024	1.000–1.048	0.046
Smoking behaviour	1.077	0.526–2.205	0.839
Anticoagulantia	1.183	0.484–2.891	0.713
Alpha blocker preop	1.914	0.699–5.238	0.206
Side: left, right, or combined	Reference 1.721 7.391	– 0.876–3.382 0.693–78.810	– 0.115 0.098
Preop JJ-cath	0.933	0.454–1.915	0.850
Postoperative catheter	1.247	0.234–6.631	0.796
Stone density	0.999	0.998–1.000	0.033
Anatomical anomaly	1.125	0.459–2.756	0.797
Stone-free	0.743	0.307–1.798	0.510
Stone size	0.973	0.868–1.092	0.646
Stone in kidney	1.032	0.486–2.192	0.935
Operative time	1.009	0.995–1.023	0.217
Operator: UIT, urologist, or combined	Reference 0.994 0.760	– 0.320–2.959 0.207–2.782	– 0.992 0.678

* *p* < 0.05, NS = not statistically significant (*p* ≥ 0.05). Abbreviations: OR, odds ratio; CI, confidence interval; BMI, body mass index; ASA, American Society of Anesthesiologists; eGFR, estimated glomerular filtration rate; preop JJ-cath, preoperative use of double J-catheter; UIT, urologist in training. Statistical test: multivariable logistic regression analysis.

## Data Availability

The data presented in this study are available on reasonable request from the corresponding author. The data are not publicly available due to privacy restrictions.
